# Study on the Chemical Composition and the Biological Activities of *Vitis vinifera* Stem Extracts

**DOI:** 10.3390/molecules27103109

**Published:** 2022-05-12

**Authors:** Talel Ben Khadher, Samir Aydi, Mohamed Mars, Jalloul Bouajila

**Affiliations:** 1 Laboratoire de Génie Chimique, Université de Toulouse, CNRS, INP, UPS, F-31062 Toulouse, France; benkhadhertalel@gmail.com; 2 Laboratory of Biodiversity and Valorization of Bioressources in Arid Zones, Faculty of Sciences, The University of Gabes, Zrig, Gabes 6072, Tunisia; samir.aydi@gmail.com (S.A.); marsmohamed@yahoo.fr (M.M.)

**Keywords:** *Vitis vinifera* stem extracts, chemical composition, biological activities, HPLC-DAD, GC-MS

## Abstract

*Vitis vinifera* (*V. vinifera*) is a herbaceous plant, cultivated worldwide and known for its biological benefits. The aim of this study is the investigation of the chemical composition as well as the determination of the biological potential of different grape stem extracts obtained by maceration and accelerated solvent extraction (ASE). The HPLC analysis of the tested extracts led to the identification of 28 compounds of which 17 were identified for the first time in grape plants, in addition to seven revealed in the stem part for the first time. Twenty-nine volatile molecules have been detected by GC-MS in the grape stem part; among them seven were identified for the first time in the grape plant. For the biological analysis, the ethyl acetate extract (EtOAc) obtained by maceration showed a significant potential regarding antioxidant activity (IC_50_ = 42.5 µg/mL), anti-Alzheimer (IC_50_ = 14.1 µg/mL), antidiabetic (IC_50_ = 13.4 µg/mL), cytotoxic with HCT-116 (IC_50_ = 12.5 µg/mL), and anti-inflammatory (IC_50_ = 26.6 µg/mL) activities, as well as showing the highest polyphenol content (207.9 mg GAE/g DW).

## 1. Introduction

In the last few years, the interest in finding bioactive compounds derived from plants has significantly increased, due to their beneficial effects on human health against various degenerative diseases, as well as their uses in many industry fields, such as pharmaceuticals, agrochemicals, flavors, fragrances, colors, biopesticides, and food additives [1]. Those compounds are usually synthesized by the plants against pathogens threats, or in case of stress conditions. They are divided into three categories: terpenoids, alkaloids, and mainly polyphenols. This class includes a large scale of molecules exerting various biological effects, such as antioxidants, antimicrobials, anti-carcinogens, and antidiabetics [2].

*V. vinifera* is an herbaceous plant cultivated in different regions of the world. Annually, there are more than 77 million tons of grape fruit produced around the world [3]. Numerous studies investigating the phytochemical composition and the biological activities of *V. vinifera* grape extracts have been carried out. Diverse health benefits have been reported, such as antioxidant, anti-cancerous, anti-bacterial, and antidiabetic effects [4]. Furthermore, grape extracts have an effective potential in the prevention of cardiovascular diseases [5], as well as exhibiting antimicrobial [6], antihypertensive [7], and anti-ulcer activities [8]. Besides the beneficial effects reported in grape extracts, studies investigating the phytochemical content of this plant have shown that grapevine extracts contain numerous bioactive molecules, such as resveratrol, caftaric, and coutaric acid, flavanol, and dihydroflavanol glycosides [9].

Recently, research into the potential uses of plant byproducts, including *V. vinifera*, has increased significantly for various reasons, including ecological concerns. In fact, the wine industry generates a considerable amount of grape waste, posing substantial environmental issues. It is estimated that grapevine by-products account for approximately 20% of the fresh harvest [10]. Diverse effects, such as antioxidant, chemopreventive, and hepatoprotective effects have been reported in different parts of *V. vinifera,* such as in the seeds, roots, leaves, and stems. Moreover, grapevine byproducts are very rich in polyphenols, such as phenolic acids; flavanols; stilbenes; quercetin, kaempferol and flavan-3-ols [11].

Grapevine stems represent 25% of the total mass of the plant waste, therefore, they are the less characterized and valued of all by-products generated [12]. Traditionally used as animal feed or as organic fertilizers [13,14]. They contain a large-scale of polyphenols, such as phenolic acids (e.g., gallic, caffeic, and caftaric acids); flavanols (e.g., quercetin-3-*O*-rutinoside, kaempferol-3-*O*-glucoside), flavanols (e.g., Epicatechin, catechin), anthocyanins (e.g., malvidin-3-*O*-glucoside, malvidin-3-*O*-(6-*O*-caffeoyl)-glucoside), stilbenes (e.g., *trans*-resveratrol, Ɛ-viniferin) and procyanidins (e.g., procyanidin B2 and B3) [1,12,13,14,15,16]. Several biological activities were reported in stem extract, such as antioxidant, anti-cancerous, antidiabetic and antibacterial activities. Nonetheless, there have been relatively few articles that studied the beneficial effects of grape stem extracts [4,17,18].

The present study aims to describe the chemical composition of *V. vinifera* stem extracts by HPLC-DAD and GC-MS as well as their biological activities mainly the antioxidant (DPPH), anti-Alzheimer (AChE), antidiabetic (Alpha amylase), anti-inflammatory (15-lipoxygenase), and anti-proliferation (HCT-116 and Caco-2) activities.

## 2. Results and Discussion

### 2.1. Extraction Yields

To the best of our knowledge, the effect of solvents on the extraction yield of *V. vinifera* stem extracts has not been reported in previous studies. Two extraction methods were used in this study: Maceration and ASE, to assess the effect of those methods on the yield extraction, the chemical composition, and the biological activities of grape stem extracts. ASE is a method that consumes much less solvent, but maceration exposes metabolites to higher pressures and temperatures than maceration. Fractional extraction was performed using organic solvents of increasing polarity (CHYA, DCM, EtOAc, MeOH, and H_2_O) and separately, an extraction with the ultrapure water (Aqueous) was performed. The extraction yield results are illustrated in (Table 1). The ASE-extracts showed higher yields values than the extracts obtained by maceration extraction, with the highest value being recorded with the aqueous extract (5.1%) while the cyclohexane extract obtained by maceration presented the lowest value (0.1%). Moreover, the yield extraction of the polar fractions is higher than that of the apolar ones. Those results let us suggest that the stem part of the studied plant is highly abundant with polar compounds, such as polyphenols than with apolar ones. In this context, a study realized by Vázquez-Armenta et al. [19] on the stem of the *V. vinifera* variety (red globe) found an extraction yield with ethanol solvent (33%) higher than our obtained results.

### 2.2. Total Polyphenol Content (TPC)

**Figure 1:**
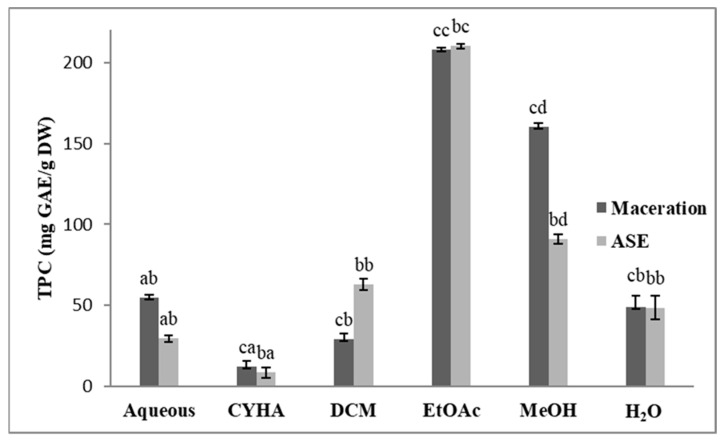
Total phenolic content (TPC) of different extracts of *V. vinifera* stem (CYHA: Cyclohexane; DCM: Dichloromethane; EtOAc: Ethyl acetate; MeOH: Methanol). Extracts were tested at 50 µg/mL Means values ± SD (*n* = 3); Different letter on the histograms means a significant difference (*p* ≤ 0.05).

The TPC of the obtained extracts was determined using the Folin method. Results are illustrated in Figure 1. Regarding the extraction method, we note a significant difference in the TPC content between the extracts of those techniques, except for the EtOAc, the MeOH, and the H_2_O ones. Concerning the solvent effect, there is a significant difference between the used solvents. The highest TPC was found in the EtOAc extract (207.9 mg GAE/g DW for the maceration extract and 210.1 mg GAE/g DW for the ASE one), while the lowest TPC was obtained in the CYHA extracts (9.8 mg GAE/g DW for the ASE extract and 14.9 mg GAE/g DW in the extract obtained by maceration. It is important to highlight that the obtained TPC is higher than that found by Vázquez-Armenta et al. [19] in the ethanolic stem extract (37.3 g GAE/kg DW) and that found by Veskoukis et al. [20] in the methanolic extract of a Greek grape stem variety (374.8 mg GAE/g DW).

### 2.3. Antioxidant Activity (DPPH)

**Figure 2:**
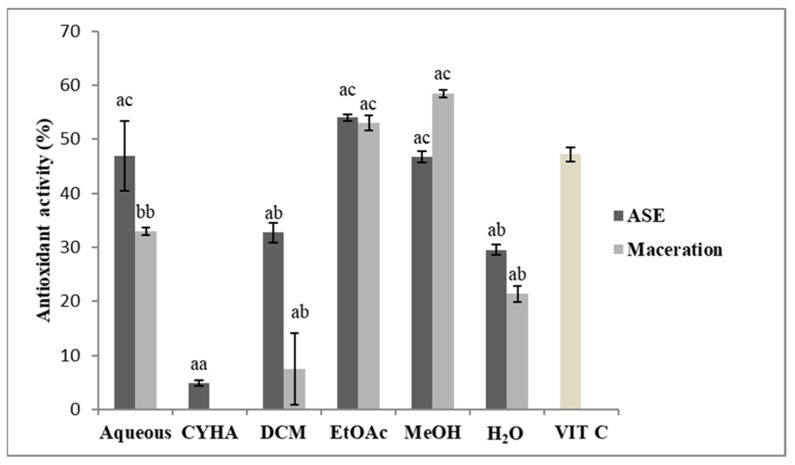
Antioxidant Activity of different extracts of *V. vinifera* stem (CYHA: Cyclohexane; DCM: Dichloromethane; EtOAc: Ethyl acetate; MeOH: Methanol; VIT C: ascorbic acid used as reference at 4 µg/mL). Extracts were tested at 50 µg/mL. Means values ± SD (*n* = 3); Different letter on the histograms means a significant difference (*p* ≤ 0.05).

The antioxidant activity of the obtained extracts was assessed using a DPPH assay. The extract concentration was adjusted to 50 µg/mL, and ascorbic acid (VIT C) was tested at 4 µg/mL, the results of inhibition activity are presented in Figure 2. Statistically, there is no significant difference between the extraction methods except for the aqueous extract obtained by maceration. Regarding the extraction solvents, the inhibition percentage is higher in the polar extracts than the apolar ones with the highest values were 53% for the EtOAc extract (IC_50_ = 43.1 µg/mL) (Table 2) and 58.5% for the MeOH extract (IC_50_ = 34.6 µg/mL) obtained by the maceration. Concerning the ASE-Extracts, the EtOAc extract exhibited 54% of inhibition activity (IC_50_ = 43.1 µg/mL), 46.9% for the aqueous extract and 46.7% for the MeOH extract. The antioxidant activity values were varied between the obtained extracts. The highest inhibitory activity was exerted mainly by the polar extracts. Those results could be explained by the correlation between TPC and the antioxidant activity of the extracts. Polyphenolic compounds are known for their antioxidant potential by scavenging reactive oxygen species [19]. Compared to the literature, the present results are less than the values found by Veskoukis et al. [20] on the three studied varieties of grape stem extract and that of the methanolic extract of Greek varieties with an average of IC_50_ = 7.8 µg/mL [21]. The comparison of the data obtained concerning radical scavenging capacity with those available in the bibliography showed significant differences [15]. This fact could be due to the distinct phenolic composition of the separate varieties as a consequence of the genetic load and agro-climatic factors [12].

### 2.4. Chromatographic Analysis

#### 2.4.1. Identification of Compounds in *V. vinifera* Stem Extracts by HPLC-DAD

**Figure 3:**
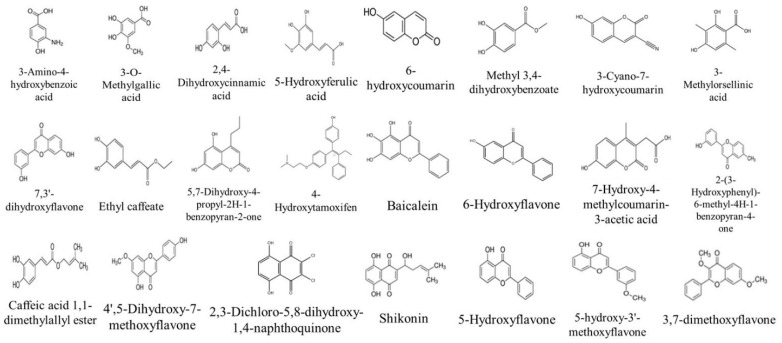
Compounds Firstly identified in *V. vinifera* Stem part by HPLC-DAD.

The identification of phenolic compounds in the different stem extracts was conducted using the HPLC-DAD method. The retention time of each peak, as well as λmax, was compared with that of standards with known retention time injected in the same conditions as the extracts. The peak area was used as a quantitative estimation of the compound. The analysis of the chemical composition of the stem extracts led to the identification of 28 compounds as presented in Table 3. Twenty-six compounds were identified for the first time in the grape stem part as presented in Figure 3, among them 17 were identified for the first time in the *V. vinifera* plant (3-Amino-4-hydroxybenzoic acid, 2,4-dihydroxycinnamic ac-id, 6-hydroxycoumarin, methyl 3,4-dihydroxybenzoate, 3-cyano-7-hydroxycoumarin, 3-methylorsellinic acid, 7,3′-dihydroxyflavone, 5,7-dihydroxy-4-propyl-2H-1-benzopyran-2-one, 4-hydroxytamoxifen (isomers E and Z), 7-hydroxy-4-methylcoumarin-3-acetic acid, 2-(3-hydroxyphenyl)-6-methyl-4H-1-benzopyran-4-one, 4′,5-dihydroxy-7-methoxyflavone, 2,3-dichloro-5,8-dihydroxy-1,4-naphthoquinone, 5-hydroxyflavone, 5-hydroxy-3′-methoxyflavone, and 3′-hydroxy-b-naphthoflavone). The majority of the identified compounds were present in the polar extracts. The EtOAc extract obtained by maceration showed a high content of compounds, such as baicalein (area = 903.5), 6-hydroxyflavone (area = 40.87) and 2,4-dihydroxycinnamic acid (area = 6.63). While the DCM extract obtained by ASE showed a high content of compounds, such as 2-(3-Hydroxyphenyl)-6-methyl-4H-1-benzopyran-4-one (area = 26.86) and Methyl 3,4-dihydroxybenzoate (area = 18.92). According to the literature, several studies have investigated the grape stem composition. Research showed that the stem is rich in various polyphenols compounds, such as gallic acid; *trans*-resveratrol and procyanidin B1 [22]. In another study, Esparza et al. [23] identified several phenolic compounds by HPLC-DAD, such as quercetin; caftaric acid and Ɛ-viniferin.

#### 2.4.2. GC-MS Analysis of the *V. vinifera* Extracts before and after Derivatization

**Figure 4:**
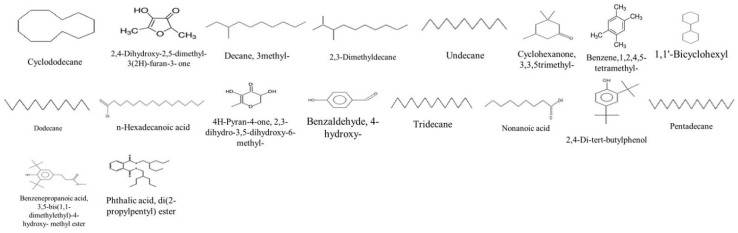
Compounds firstly identified in *V. vinifera* Stem part by GC-MS (before derivatization).

**Figure 5:**
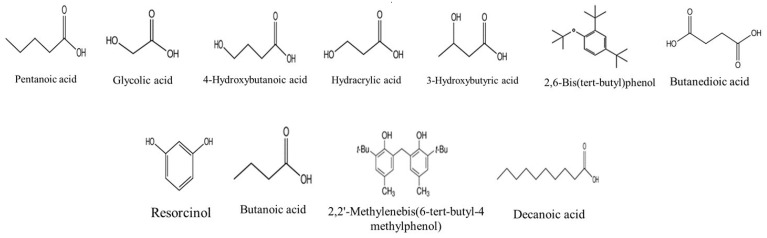
Compounds firstly identified in *V. vinifera* Stem part by GC-MS (after derivatization).

The volatile composition of the different stem extracts was determined by using the GC-MS method. The results of the identification of the compounds were presented in Table 4 and Table 5. As shown in the tables below. The GC-MS technique has led to the identification of 36 compounds (before and after derivatization). The compounds identified for the first time are shown in Figure 4 and Figure 5. Seven compounds were identified for the first time in the grape plant: (decane, 3-methyl-, 2,3-dimethyldecane, pentadecane, phthalic acid, di(2-propylpentyl) ester, hydracrylic acid, nonanoic acid, and 2,2′-methylenebis(6-tert-butyl-4-methylphenol) several compounds were in common between the two extraction methods, such as cyclododecane, decane-3-methyl-, and undecane. In contrast, organic acid compounds, such as pentanoic acid, hexanoic acid, butanedioic acid, and glycolic acid were abundant in extracts after derivatization. Moreover, other class compounds were detected in the stem extracts, such as furanones (e.g., 2,4-dihydroxy-2,5-dimethyl-3(2H)-furan-3-one), phenolic (e.g., resveratrol, gallic acid). Most of the identified volatile compounds were detected in the maceration extracts, mainly the apolar ones due to their affinity, therefore, few were revealed in the fractions obtained by ASE. As shown in Table 4 and Table 5, some compounds were present in two or three extracts, e.g., 2,4-dihydroxy-2,5-dimethyl-3(2H)-furan-3-one. This is due to their release from the disrupted cells of the plant material during the maceration at room temperature. According to the literature, few studies have investigated the volatile profile of the stem part. In the same context, Matarese et al. [48] identified several volatile compounds in the stem part; monoterpenes, such as (E)-β-ocimene, 1,1-dimethyl-3-methylene-2-vinylcyclohexane, limonene and camphene; sesquiterpenes, such as α-farnesene, β-caryophyllene and α-humulene; benzenic compounds, such as benzaldehyde, 2-acetyl-4-methylphenol and methyl salicylate and aliphatic compounds, such as 2,4-hexadienal, (E,E)-, 1-octen-3-ol and nonanal.

### 2.5. Biological Activities

#### 2.5.1. Anti-Inflammatory Activity

**Figure 6:**
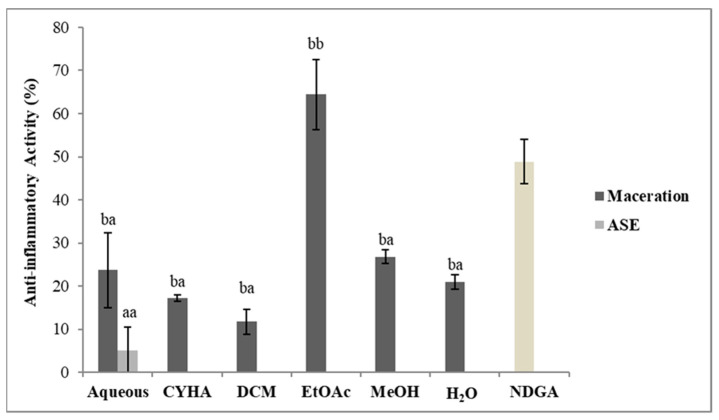
Anti-15-lipoxygenase activity of different extracts of *V. vinifera* stem (CYHA: Cyclohexane; DCM: Dichloromethane; EtOAc: Ethyl acetate; MeOH: Methanol; NDGA: nordihydroguaiaretic acid used as reference). Extracts were tested at 50 µg/mL; NDGA was tested at 4 µg/mL. Means values ± SD (*n* = 3); Different letter on the histograms means a significant difference (*p* ≤ 0.05).

The anti-inflammatory activity of the obtained extracts was conducted at 50 µg/mL using the 15-lipoxygenase assay. Nordihydroguaiaretic acid (NDGA) at 4 µg/mL was used as a reference in this activity. The results of the inhibition activity are shown in Figure 6. Statistically, there is a difference between the extraction methods. All maceration extracts have shown inhibition against the lipoxygenase enzyme; the highest value was 64.5% in the EtOAc extract (IC_50_ = 26.6 µg/mL) while only the aqueous extract was obtained by ASE induced a slight inhibition of 5%. The variation in the inhibition activity between the two methods especially with the EtOAc extract could be due to degradation or structural changes in the compounds responsible for this activity due to the high temperature and pressure used in the ASE extraction. Moreover, the EtOAc obtained by maceration has shown a high inflammatory effect with an IC_50_ 6.4 times less effective than that of the NDGA (4 µg/mL). Nevertheless, the EtOAc activity was significantly high and resulted from the presence of bioactive compounds, such as Baicalein and resveratrol (Table 3 and Table 5), known for their anti-inflammatory effects. In the same context, according to the literature, the stem as the other part of the *V. vinifera* plant was known for its anti-inflammatory potential. In the same context, Leal et al. [22] proved that the MeOH extracts of *V. vinifera* stem varieties presented anti-inflammatory capacities, exhibiting inhibitions of nitric oxide production, by lipopolysaccharide-stimulated macrophages, up to 35.3%.

#### 2.5.2. Anti-Alzheimer Activity

**Figure 7:**
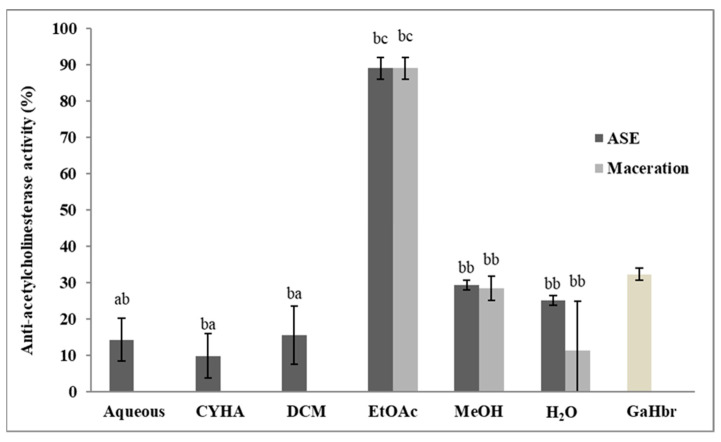
Anti-Alzheimer activity of different extracts of *V. vinifera* stem (CYHA: Cyclohexane; DCM: Dichloromethane; EtOAc: Ethyl acetate; MeOH: Methanol; GaHbr: Galantamine Hydrobromide used as reference at 1.5 µg/mL). Extracts were tested at 50 µg/mL Means values ± SD (*n* = 3); Different letter on the histograms means a significant difference (*p* ≤ 0.05).

The anti-Alzheimer activity of stem extracts was performed according to the acetylcholinesterase assay. Galanthamine hydrobromide (GaHbr) tested at 1.5 µg/mL was used as a reference and the different extracts were tested at 50 µg/mL. As shown in Figure 7, the extracts obtained by maceration and by ASE inhibited the AChE enzyme with some differences. The totality of the ASE extracts has shown activity while only three extracts by maceration were conducted (EtOAc, MeOH, and H_2_O). The highest inhibition value was recorded with EtOAc extract by the two methods, 94.8% (IC_50_ = 14.1 µg/mL) for the extract obtained with maceration and 89% (IC_50_ = 18.7 µg/mL). Those results suggest that phenolic compounds, such as resveratrol and its derivatives could be responsible for this activity. This suggestion could be justified by the previous studies that reported the anti-Alzheimer effects of several phenolic compounds, in addition to the high correlation between TPC and anti-Alzheimer activity (r = 0.912) (Table 6). In the same context, Papastamoulis et al. [78] isolated Viniphenol A, a Complex resveratrol hexamer from *V. vinifera* stem extract that showed neuroprotective effects against the amyloid-β peptide (Aβ)-induced neurotoxicity in cultured PC12 cells using the MTT assay.

#### 2.5.3. Cytotoxic Activity

**Figure 8:**
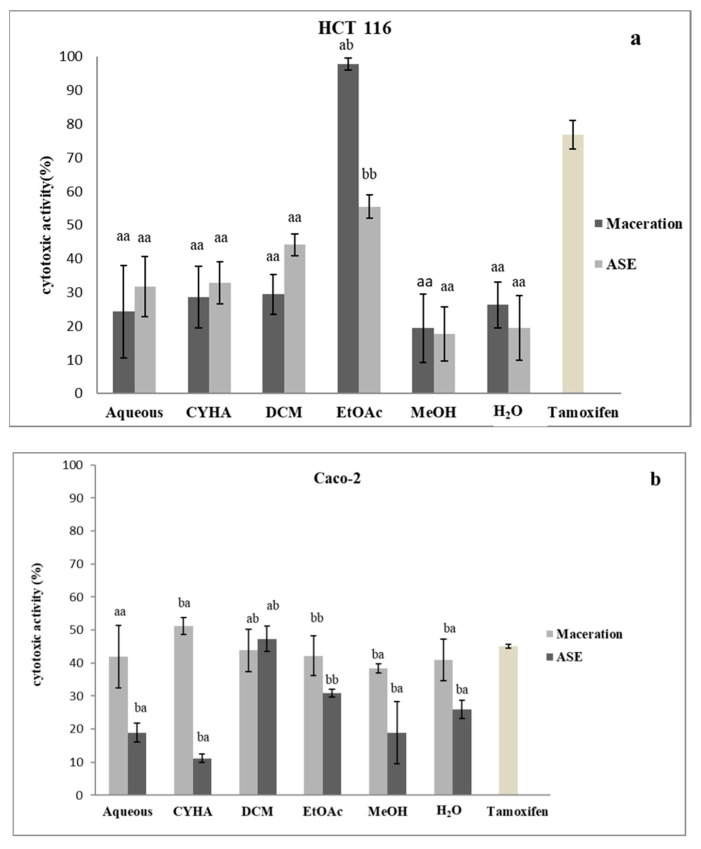
Cytotoxic activity of different extracts of *V. vinifera* stem against two cell types (HCT 116: (**a**); CaCo-2: (**b**)). (CYHA: Cyclohexane; DCM: Dichloromethane; ETOAC: Ethyl acetate; MeOH: Methanol; Tamoxifen: used as referance at 100 µM). Extracts were tested at 50 µg/mL. Means values ± SD (*n* = 3); Different letter on the histograms means a significant difference (*p* ≤ 0.05).

In this study, the determination of the cytotoxic activity of the grape stem extracts was conducted using the MTT method against two cancer cell lines (HCT-116 and Caco-2). The extracts were adjusted at 50 µg/mL and tamoxifen was used as a reference. The results of the inhibition activity are shown in Figure 8. Statistically, there was a significant difference between the extraction methods, as well as with the extraction solvents. Firstly, for the HCT-116 cell line, the highest inhibition activity was detected for the ethyl acetate extract obtained at 97.9% by maceration (IC_50_ = 12.5 µg/mL) and 55.5% by ASE, the other extracts exhibited moderate to low inhibition activity, with the highest being the DCM extract by ASE, with 44.2% of inhibition activity. For the second cell line, the inhibition activity exhibited by the majority of the extracts was moderate to low with the highest value recorded for the CHYA extract obtained by maceration with 51.2%, and for the ASE technique, the DCM extract with 47.3%. The variation in the cytotoxic activity of extracts can be due to various reasons, such as the specificity of the chemical composition of each extract, or the difference in the mechanism of lysis of the cancer cell. In the same context, a study conducted by Amico et al. [79] on the antiproliferative effect of the stem extract from a grape of the Sicilian *V. vinifera* variety ‘Nerello Mascalese’ has shown that ethyl acetate extract inhibits MCF7 with 62% at 1000 µg/mL. In other research, stem extracts from four Greek varieties were tested against four cell lines. The obtained results were lower than the IC_50_ of the ethyl acetate extract against the HCT-116 cell, and the values of IC_50_ were 121–230 (MCF-7), 121–184 (MDA-MD-23), 175–309 (HT29), 159–314 (K1), 180–225 (786-O) and 134–>400 μg/mL (Caki-1) [14]. 

#### 2.5.4. Antidiabetic Activity

**Figure 9:**
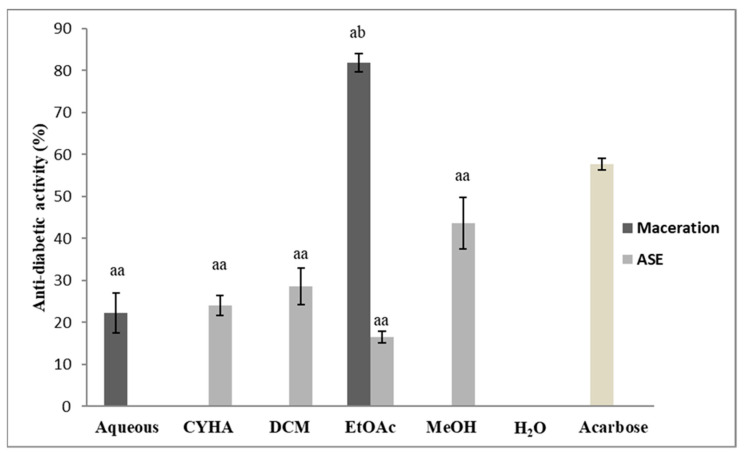
Antidiabetic Activity of different extracts of *V. vinifera* stem (CYHA: Cyclohexane; DCM: Dichloromethane; EtOAc: Ethyl acetate; MeOH: Methanol; Acarbose: used as reference at 50 µg/mL). Extracts were tested at 50 µg/mL Means values ± SD (*n* = 3); Different letter on the histograms means a significant difference (*p* ≤ 0.05).

The determination of the antidiabetic potential of the grape stem extracts was established using the alpha-amylase assay. Extracts were prepared at 50 µg/mL, acarbose was used as a positive standard. The results of the antidiabetic activity are presented in Figure 9. Statistically, there is no significant difference between the extractions methods as well as between the extraction solvents except for the ethyl acetate. The EtOAc extract obtained by maceration was the only one that exerted a high inhibition activity among all the tested extracts. It inhibits amylase activity with 81.9% (IC_50_ = 13.4 µg/mL); for the extracts obtained by ASE only the MeOH extract presented a moderate activity, with 43.6%. The variation in the results could be due to the difference in the extraction conditions (temperature, pressure; extraction time) as well as the affinity of the bioactive compounds to the extraction solvents. In the same context, a study on the antidiabetic activity of grape stem extract, conducted by Ahmed et al. [80] proved that the chloroform and the ethanolic stem extract reduced blood glucose levels in a dose-dependent manner with the highest activity at 200 mg/kg bodyweight concentration.

### 2.6. Principal Component Analysis (PCA)

**Figure 10:**
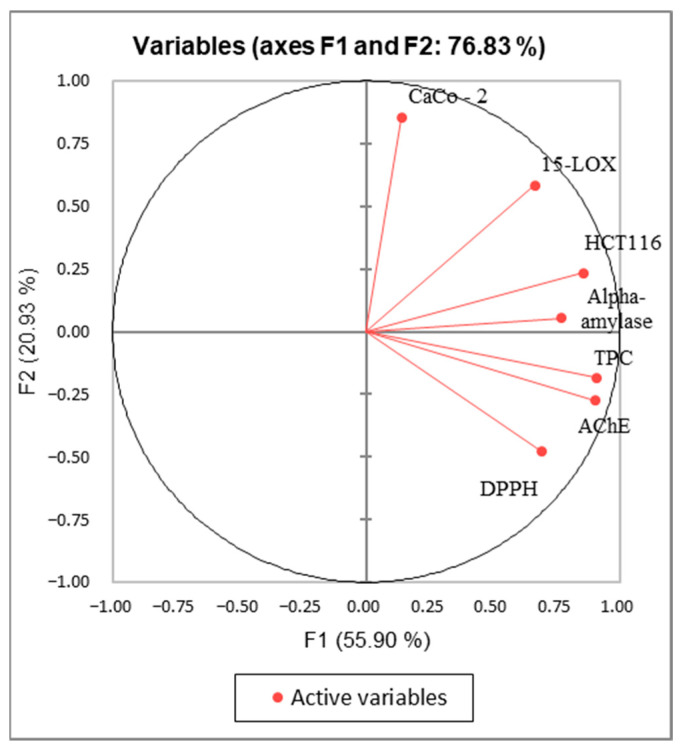
Principal Components analysis “loading plot” of (DPPH: Antioxidant activity) (TPC: total phenolic content) and biological activities (Alpha amylase: Antidiabetic activity); 15-LOX: Anti-inflammatory activity); (HCT-116 and Caco-2: cytotoxic activity) and (AChE: anti-Alzheimer activity) of *V. vinifera stem* extracts.

**Figure 11:**
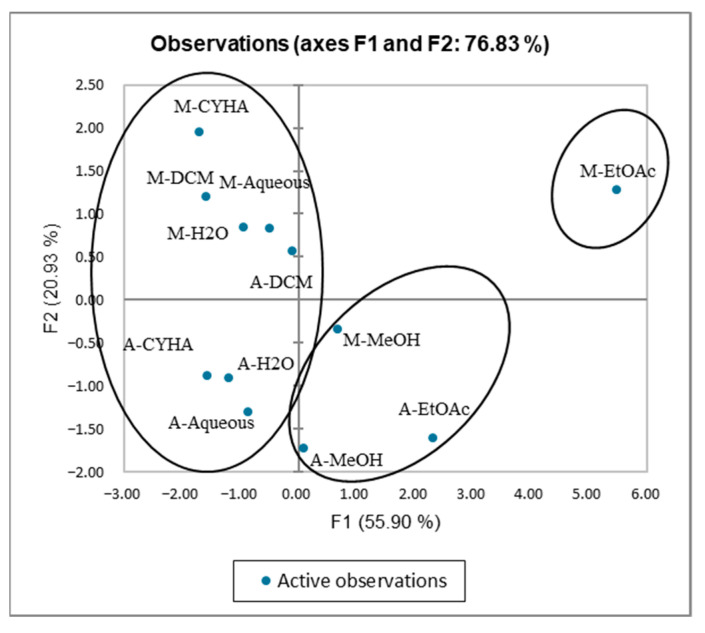
Principal Components analysis “score plot” of (DPPH: Antioxidant activity) (TPC: total phenolic content) and biological activities (Alpha amylase: Antidiabetic activity); 15-LOX: Anti-inflammatory activity); (HCT-116 and Caco-2: cytotoxic activity) and (AChE: anti-Alzheimer activity) of *V. vinifera* stem extracts.

**Figure 12:**
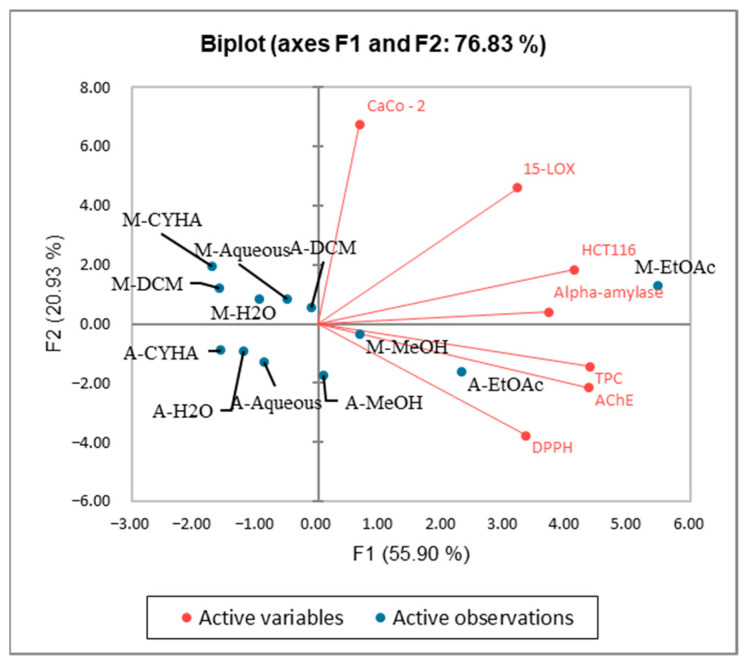
Principal Components analysis “Biplot” of (DPPH: Antioxidant activity) (TPC: total phenolic content) and biological activities (Alpha amylase: Antidiabetic activity); 15-LOX: Anti-inflammatory activity); (HCT-116 and Caco-2: cytotoxic activity) and (AChE: anti-Alzheimer activity) of *V. vinifera* stem extracts.

The Kaiser–Meyer–Olkin (KMO) test was performed to assess the use of factorial analysis and PCA for the studied variables in this research. The KMO index obtained is 0.549 > 0.5, indicating that this test is suitable for the data of the study. The PCA analysis was performed to establish the correlation between the TPC and the different biological activities. The axes of inertia have been withheld from this analysis. The percentage of the total variation was recorded at 76.83%. The PC1 and the PC2 axes expressed 55.9% and 20.9% of variability as presented in Figure 10. The loadings in the PCA loading plot express, simultaneously, the correlation of the principal components with the original variables, and the correlations between the different activities and TPC. PC1 was well correlated with TPC, DPPH, anti-inflammatory, antidiabetic, and anti-Alzheimer activities, and the cytotoxic activity against the HCT-116 cell line with loading of 0.906, 0.694, 0.665, 0.771, 0.906, and 0.857, respectively. The PC2 was in positive correlation with the cytotoxic activity against Caco-2 with loading of 0.853 as mentioned in Table 7. Based on Figure 10 and the correlation matrix (Table 6), we can observe that there is a good correlation between TPC, DPPH, HCT-116, and AchE, which suggest that the phenolic compounds present in stem extracts could be responsible for those activities. A large scale of phenolic compounds has been identified, such as resorcinol and resveratrol which are known for their beneficial activities. The alpha-amylase, 15-LOX, in addition to the cytotoxic activity (HCT-116 and CaCo-2) seemed somehow correlated. This could be explained by the presence of bioactive compounds in the extracts responsible for those activities. The oval forms grouped the different extracts into three classes as shown in Figure 11; C1 (M-MeOH, A-EtOAc, and A-MeOH), C2 (M-EtOAc), and C3 (A-aqueous; M-aqueous, A-CHYA, M-CHYA, M-DCM, A-DCM, A-H_2_O, and M-H_2_O). The correlation between variables and observations was shown in Figure 12. The M-EtOAc extract is near TPC, AChE, alpha-amylase, and HCT-116. This correlation is related to the phenolic composition present in this extract that let us suggest compounds, such as Baicalein, resorcinol, and resveratrol.

## 3. Materials and Methods

### 3.1. Plant Material

The sample used in this study was the stem of the white Sauvignon variety of the *V. vinifera* species (France). After air-drying, the collected plant material was ground (LGC France) using a Lescha super-ZAK grounder Hamburg (Germany) and stored at room temperature.

### 3.2. Extraction

Two methods were used to extract the secondary metabolites from the collected stem: maceration and ASE. The purpose of using organic solvents was to deplete the plant matter of their metabolites and to split them according to polarity.

#### 3.2.1. Maceration

One hundred grams of dried sample were successively extracted with organic solvents of increasing polarity: CYHA, DCM, EtOAc, MeOH, and H_2_O for 2 h under medium agitation at ambient pressure and temperature. The mixture was filtered using Wattman paper and the solvents were evaporated using a rotary evaporator under a vacuum at 35 °C (IKA, RV 10 auto V, Staufen Germany). Separately 50 g of the same sample was extracted with water under the same conditions described above.

#### 3.2.2. Accelerated Solvent Extraction (ASE)

ASE was performed by the Dionex extraction system (ASE 100, Sunnyvale, CA, USA). for the fractioned extraction, the extraction cell was filled with 30 g of the sample using, respectively, the organic solvents CYHA, DCM, EtOAc, MeOH, and H_2_O for the aqueous extraction, the same weight was extracted using the water. The extraction conditions were as follows, the temperature was 80 °C; the pressure was 110 bar. Nitrogen was used as a purge gas. The obtained extract was collected in a 250 mL collection bottle then the solvent was evaporated using a rotary evaporator under a vacuum at 35 °C (IKA, RV 10 auto V, Staufen Germany).

### 3.3. Total Phenolic Content (TPC)

The TPC of the obtained extracts was determined using the Folin–Ciocalteu method as described by Rahmani et al. [81] with some modification. Briefly, In a 96-well microplate, 20 µL of each plant extract (1 mg/mL) was mixed with 100 µL of Folin–Ciocalteu reagent (0.2 N). After 5 min of incubation at 25 °C, 80 µL of Na_2_CO_3_ (75 g/L) was added then the reaction mixture was reincubated for 15 min. The absorbance was measured at 765 nm using a microplate reader (Multiskan Go, F1-01620, Thermo Fisher Scientific, Vantaa, Finland). A standard calibration curve was plotted using Gallic acid (0 to 115 mg/L). Results were expressed as mg of Gallic acid equivalents (GAE)/g DW.

### 3.4. Determination of DPPH Radical Scavenging Activity

The antioxidant scavenging activity was studied using 1,1-diphenyl-2-picrylhydrazyl free radical (DPPH), as described by Blois, [39] with some modifications. In a 96-well microplate (Micro Well, Thermo Fisher Scientific, Illkirch France), 20 μL of the diluted plant extract (0.5 mg/mL) was added to 180 μL of 0.2 mM methanolic DPPH solution. The reaction mixture was incubated at 25 °C for 25 min, then the absorbance was measured at 524 nm using a microplate reader (Multiskan Go, F1-01620, Thermo Fisher Scientific, Vantaa, Finland). Ascorbic acid (4 µg/mL) was used as the positive control. The DPPH inhibition was calculated as % inhibition = 100 × (A_blank_ − A_sample_)/A_blank_. The A_blank_ was measured without extract.

### 3.5. Biological Activity

#### 3.5.1. Anti-Inflammatory Activity

The anti-inflammatory activity of the *V. vinifera* stem extracts was determined with soybean lipoxygenase as described by Rahmani et al. [81] with some modifications. The activity measurement was tested in a 96-well plate containing: 20 µL of the plant extract (625 mg/L), 150 μL of 100 mM phosphate buffer (pH 7.4), 60 μL of linoleic acid, and 20 µL of 5-LOX enzyme solution. The mixture was then incubated at 25 °C for 10 min and the absorbance was measured at 234 nm. NDGA (0.5 mg/mL) was used as a standard. The enzyme activity inhibition was calculated as follows: % inhibition = 100 × (A_blank_ − A_sample_)/A_blank_.

#### 3.5.2. Anti-Cholinesterase Activity

The anti-cholinesterase activity of the plant extracts was performed using Ellman’s method, as previously reported by Bekir et al. [82] with some modifications. In a 96 well microplate, 50 µL of sodium phosphate buffer (0.1 mM, pH = 8) was added to 25 µL of plant extract (0.5 mg/mL), 125 µL of DTNB and 25 µL of AchE solution. The reaction mixture was incubated at 25 °C for 15 min then 25 µL of acetylthiocholine iodide substrate is added. After 10 min of incubation at 25 °C, the hydrolysis of acetylthiocholine iodide was monitored by the formation of the yellow 5-thio-2-nitrobenzoate anion; the result of the reaction of DTNB with thiocholines, was catalyzed by enzymes at a wavelength of 412 nm. GaHbr at 15 mg/L was used as a positive control. The enzyme activity inhibition was calculated as follows: % inhibition = 100 × (A_blank_ − A_sample_)/A_blank_.

#### 3.5.3. Anti-α-Amylase Activity

The anti-α-amylase activity of the plant extracts was measured by the DNS method as described by Premakumara et al. [83]. With modifications, in this practice, a reaction blend containing 25 µL of the plant extract (1.3 mg/mL) and 25 µL of enzyme solution (2 mg/mL) were incubated at 25 °C for 10 min, and then 50 µL of starch solution 1% was added to the reaction mixture. After 3 min the enzyme reaction was terminated by adding 50 µL of 3,5-dinitrosalicylic acid (96 mM). The mixture was then boiled for 10 min in a water bath and then cooled at ambient temperature and 500 µL of sodium phosphate buffer (0.1 mM) was added. The α-amylase-inhibitory activity was measured at 530 nm using a Multiskan Go microplate reader (Thermo Fisher Scientific, Vantaa, Finland). Acarbose (1.3 mg/mL) was used as a positive control. The enzyme activity inhibition was calculated as follows: % inhibition = 100 × (A_blank_ − A_sample_)/A_blank_.

#### 3.5.4. Cytotoxic Activity

Cytotoxic activity of the obtained extracts was estimated on the human colon cancer cell line, HCT 116 and the human colon adenocarcinoma cell line, Caco-2 (American Type Culture Collection, Manassas, VA, USA) as described by Rahmani et al. [81] with some modifications. In a 96-well microplate, cells were distributed at 13 × 10^3^ cells/well for HCT116 and 12 × 10^3^ cells/well for Caco-2 in 100 µL. After 24 h of incubation at 37 °C, 100 µL of each extract diluted in the medium after being solubilized in DMSO was added to 100 µL of the corresponding culture medium; RPMI (RPMI 1640, Thermo Fisher Scientific, Illkirch France) for HCT-116, or DMEM (Advanced DMEM, Thermo Fisher Scientific). The plate was then incubated for 48 h at 37 °C and the cytotoxic potential of the tested samples was evaluated by the 3-(4, 5-dimethylthiazol-2-yl)-2,5-diphenyltetrazolium bromide (MTT) assay. After removing the supernatant, cells were treated with 50 µL of MTT solution, and the plate was incubated for 40 min at 37 °C; then MTT was eliminated and 80 µL of DMSO was added. The absorbance was measured at 605 nm using a microplate reader (Multiskan Go, F1-01620, Thermo Fisher Scientific, Vantaa, Finland). Tamoxifen at 100 µM was used as a positive control.

### 3.6. Chromatographic Analysis

#### 3.6.1. High-Performance Liquid Chromatography Analysis (HPLC-DAD)

The HPLC analysis of the different extracts was performed in a Thermo Scientific Spectra SYSTEM P1000XR pump equipped with a PDA detector Waters 996. Separation was accomplished using an RP-C18 column (Phenomenex, Le Pecq, France) with dimensions of 25 cm × 4.6 mm and particle size of 5 µm. Elution was carried out at a flow rate of 1.2 mL/min with a mobile phase made up of acidified water (pH = 2.65) as Solvent A and acidified water/ACN (20:80 v/v) as solvent B. The samples were eluted by the following linear gradient: from 12% B to 30% B for 35 min, from 30% B to 50% B for 5 min, from 50% B to 99.9% B for 5 min, and finally from 99.9% B to 12% B for 15 min. Extracts were prepared at the concentration of 20 mg/mL using acidified water/ACN (80:20 *v*/*v*) and filtered using a filter (Sigma Aldrich, Millex-HA filter 0.45 µm, Saint-Quentin fallavier, France). The identification of the compounds was accomplished by comparing them with the retention time of some known standards.

#### 3.6.2. Gas Chromatography-Mass Spectrometry (GC-MS) Analysis

The analysis of the volatile profile of the Grape stem extracts was carried out using the method mentioned Rahmani et al. [81] with slight modifications. Extracts were dissolved in their extraction solvents (except for water extract, where methanol was used) at 5 mg/mL. Saturn 2000 Gas Chromatography (Les Ulis, France) was used in this analysis. Chromatographic conditions were 60 °C held for 1 min, up to 260 °C at a gradient of 5 °C/min, then hold for 15 min at 260 °C. A second gradient was applied to reach 340 °C at 40 °C/min. The trap temperature was 250 °C and that of the transfer line was 270 °C. Mass scanning was performed from 70 to 650 *m*/*z*; 2 μL of each extract was injected.

- Derivatization method:

The derivatization method was that described by Rahmani et al. [84] with some modifications. Extracts were solubilized in acetonitrile at 5 mg/mL. Then 60 µL of *N*,*O*-bis(trimethylsilyl)trifluoroacetamide (BSTFA) reagent was added to 340 µL of each extract. The reaction mixture was incubated at 40 °C for 30 min; 2 μL of each derivative solution was injected into the GC-MS and analyzed as described in the previous section.

- Compounds identification:

The identification of volatile compounds by GC-MS of grapevine stem extracts has been performed by using the Xcalibur software version 3.0.63 by comparison of their mass spectra with those recorded in the Nist MS library version 2.4 build 25 March 2020.

### 3.7. Statistical Analysis

All data were expressed as means ± standard deviations of triplicate measurements. The confidence limits were set at *p* < 0.05 calculated according to the ANOVA test using the Statistical Package for the Social Sciences (SPSS) 22 (Version IBM. 22.0. 2013, San Francisco, CA, USA, www.ibm.com (accessed on 15 January 2022)). The difference between the used solvents and extraction methods was estimated by Tukey’s test. Principal component analysis (PCA) was also conducted using XLSTAT (version 2021.3.1, Addinsoft, Pearson edition, Waltham, MA, USA).

## 4. Conclusions

The present study gives new insights into the biological activities as well as the chemical composition of *V. vinifera* stem extracts. The HPLC-DAD analysis allowed for the identification of 17 compounds not previously detected in the grape plant. The GC-MS has led to the identification of 36 compounds of which 29 were revealed for the first time in the grape stem part. Moreover, the biological investigation of the obtained extracts showed that EtOAc extract obtained by maceration has significant potential compared to most of the tested assays: antioxidant (IC_50_ = 42.5 µg/mL), anti-Alzheimer (IC_50_ = 14.1 µg/mL), antidiabetic (IC_50_ = 13.4 µg/mL), cytotoxic with HCT-116 (IC_50_ = 12.5 µg/mL), and anti-inflammatory (IC_50_ = 26.6 µg/mL), as well as showing the highest polyphenol content (207.9 mg GAE/g DW). The obtained findings encourage us to search for the bioactive compounds that are responsible for biological activities by using advanced fractionation techniques.

## Figures and Tables

**Table 1:** Extraction yields of *V. vinifera* stem extracts (DW %) extracts (CYHA: cyclohexane, DCM: dichloromethane, EtOAc: ethyl acetate, MeOH: methanol).

		Fractional Extraction				
	Aqueous	CYHA	DCM	EtOAc	MeOH	H_2_O
ASE	5.08	0.11	0.20	0.31	2.24	2.91
Maceration	3.05	0.07	0.17	0.25	1.94	3.23

**Table 2:** IC_50_ values (µg/mL) of active extracts of *V. vinifera* Stem.

Extract/Activity	Antioxidant	Anti-Inflammatory	Antidiabetic	Cytotoxic (HCT 116)	Anti-Alzheimer
EtOAc (Maceration)	42.5 ± 2.9	26.6 ± 6.6	13.4 ± 0.3	12.5 ± 1.3	14.1 ± 1.0
EtOAc (ASE)	43.1 ± 2.8	>50	>50	>50	18.7 ± 0.7
MeOH (Maceration)	34.6 ± 0.8	>50	>50	>50	>50

**Table 3:** Identification of compounds by HPLC-DAD of the grape stem extracts (A: Aqueous, C: cyclohexane, D: dichloromethane, E: ethyl acetate, M: methanol, H: H_2_O.

		% Area												
Compounds	RT	Maceration						ASE						Ref.
			Fractional Extraction						Fractional Extraction					
		Aqueous	CYHA	DCM	EtOAc	MeOH	H_2_O	Aqueous	CYHA	DCM	EtOAc	MeOH	H_2_O	
3-Amino-4-hydroxybenzoic acid	2.2	0.54	0.10	0.15		0.99	0.71							[24]
Gallic acid	3.5	1.34			0.81	0.59	0.73	0.56			0.58	0.28	0.46	[25]
3-*O*-Methylgallic acid	7.7						0.21					8.28		[26]
(−)-Epicatechin	11.9		0.19			0.02				0.02				[27]
2,4-Dihydroxycinnamic acid	14.5				6.63			0.19						[28]
5-Hydroxyferulic acid	14.5						0.14	1.07	0.06					[29]
6-hydroxycoumarin	18.2							2.24						[30]
Methyl 3,4-dihydroxybenzoate	19.2									18.92	11.09			[31]
Rutin hydrate	22.7	0.25						6.92						[32]
3-Cyano-7-hydroxycoumarin	30.3	0.09				3.97						3.14		[33]
3-Methylorsellinic acid	35.2					1.35	0.02							[34]
*Trans*-Cinnamic acid	41.5				0.25									[35]
7,3′-dihydroxyflavone	41.9				1.07	0.15	0.04				4.32			[36]
Ethyl caffeate	42							1.51				4.34		[37]
5,7-Dihydroxy-4-propyl-2H-1-benzopyran-2-one	42.8	0.02												[38]
4-Hydroxytamoxifen	43.1									1.71				[39]
Baicalein	44.5		0.47		903.45	121.22	7.73							[40]
6-Hydroxyflavone	45				40.87			5.27				2.37		[41]
7-Hydroxy-4-methylcoumarin-3-acetic acid	45.5										21.43			[42]
2-(3-Hydroxyphenyl)-6-methyl-4H-1-benzopyran-4-one	45.7	0.03								26.86				[43]
Caffeic acid 1,1-dimethylallyl ester	46												8.55	[41]
4′,5-Dihydroxy-7-methoxyflavone	46.3						0.05							[44]
3,7-dimethoxyflavone	47.5			0.03										[45]
2,3-Dichloro-5,8-dihydroxy-1,4-naphthoquinone	47.7			0.59										[46]
Shikonin	48								0.15	0.92				[47]
5-Hydroxyflavone	48.5							0.36						[44]
5-Hydroxy-3′-methoxyflavone	49.4	3.03					0.83	0.42	0.36				0.07	[44]
3′-Hydroxy-b-naphthoflavone	49.8												0.083	[44]

**Table 4:** Identification of volatile compounds by GC-MS of the extracts of *V. vinifera* stem (before derivatization) (CYHA: cyclohexane, DCM: dichloromethane, EtO: ethyl acetate, M: methanol, H: H_2_O.

No.	RI	Compound	Maceration						ASE						Ref.
				Fractional Extraction						Fractional Extraction					
			Aqueous	CYHA	DCM	EtOAc	MeOH	H_2_O	Aqueous	CYHA	DCM	EtOAc	MeOH	H_2_O	
1	1019	Cyclododecane	X			X						X			[49]
2	1044	2,4-Dihydroxy-2,5-dimethyl-3(2H)-furan-3-one	X				X	X	X						[50]
3	1075	Decane, 3-methyl-		X											[51]
4	1088	2,3-Dimethyldecane								X					[52]
5	1100	Undecane		X						X					[53]
6	1119	Cyclohexanone, 3,3,5trimethyl-				X									[50]
7	1187	Benzene, 1,2,4,5-tetramethyl-			X										[54]
8	1205	Dodecane								X					[50]
9	1249	4H-Pyran-4-one, 2,3-dihydro-3,5-dihydroxy-6-methyl-	X			X	X	X					X	X	[55]
10	1306	Tridecane		X						X					[56]
11	1312	Nonanoic acid		X											[56]
12	1404	1,1′-Bicyclohexyl								X					[57]
13	1470	Benzaldehyde, 4-hydroxy-	X		X	X									[58]
14	1533	Pentadecane		X											[59]
15	1599	2,5-di-tert-Butyl-1,4-benzoquinone			X										[60]
16	1617	2,4-Di-tert-butylphenol	X	X	X										[61]
17	2161	n-Hexadecanoic acid	X	X	X	X									[62]
18	2194	Benzenepropanoic acid, 3,5-bis(1,1-dimethylethyl)-4-hydroxy- methyl ester	X			X									[63]
19	2717	Phthalic acid, di(2-propylpentyl) ester	X												[64]

X: detected.

**Table 5:** Identification of volatile compounds by GC-MS of the extracts of *V. vinifera* stem (with derivatization) ) (CYHA: cyclohexane, DCM: dichloromethane, EtOAc: ethyl acetate, MeOH: methanol).

No.	RI	Compound	Maceration						ASE						Ref.
				Fractional Extraction						Fractional Extraction					
			Aqueous	CYHA	DCM	EtOAc	MeOH	H_2_O	Aqueous	CYHA	DCM	EtOAc	MeOH	H_2_O	
1	1013	Pentanoic acid								X	X				[65]
2	1094	Hexanoic acid			X	X	X				X				[66]
3	1112	Glycolic acid								X					[66]
4	1187	Hydracrylic acid										X			[67]
5	1199	3-Hydroxybutyric acid										X			[68]
6	1283	4-Hydroxybutanoic acid									X				[69]
7	1377	Butanedioic acid								X	X	X			[70]
8	1436	Resorcinol				X	X				X				[71]
9	1517	Decanoic acid								X					[72]
10	1636	2,6-Bis(tert-butyl)phenol							X	X					[73]
11	1710	Tartaric acid								X	X	X			[74]
12	1926	2,2′-Methylenebis(6-tert-butyl-4 methylphenol)	X						X						[75]
13	2043	Pentadecanoic acid								X					[76]
14	2064	Palmitic Acid		X	X	X				X	X	X			[76]
15	2074	Gallic acid										X			[21]
16	2128	Resveratrol												X	[21]
17	2342	9,12-Octadecadienoic acid (Z,Z)-								X					[77]

X: detected.

**Table 6:** Correlation matrix (Pearson (*n*)).

Variables	DPPH	HCT116	CaCo-2	AChE	15-LOX	Alpha-Amylase	TPC
DPPH	**1**	0.316	−0.153	**0.671**	0.247	0.366	**0.792**
HCT116	0.316	**1**	0.212	**0.763**	**0.624**	**0.744**	**0.627**
CaCo-2	−0.153	0.212	**1**	−0.074	0.472	−0.028	0.113
AChE	**0.671**	**0.763**	−0.074	**1**	0.369	**0.594**	**0.912**
15-LOX	0.247	**0.624**	0.472	0.369	**1**	0.527	0.470
Alpha-amylase	0.366	**0.744**	−0.028	**0.594**	0.527	**1**	0.517
TPC	**0.792**	**0.627**	0.113	**0.912**	0.470	0.517	**1**

Values in bold are different from 0 with a significance level alpha = 0.05.

**Table 7:** Correlations between variables and factors.

	F1	F2
DPPH	0.694	−0.480
HCT116	0.857	0.234
CaCo-2	0.138	0.853
AChE	0.906	−0.274
15-LOX	0.665	0.583
Alpha-amylase	0.771	0.053
TPC	0.906	−0.183

## Data Availability

The study did not report any data.
